# Structures of cyanobacterial bicarbonate transporter SbtA and its complex with PII-like SbtB

**DOI:** 10.1038/s41421-021-00287-w

**Published:** 2021-08-10

**Authors:** Xiao-Yu Liu, Wen-Tao Hou, Liang Wang, Bo Li, Yu Chen, Yuxing Chen, Yong-Liang Jiang, Cong-Zhao Zhou

**Affiliations:** Hefei National Laboratory for Physical Sciences at the Microscale and School of Life Sciences, University of Science and Technology of China, Hefei, Anhui China

**Keywords:** Cryoelectron microscopy, Plant molecular biology

Dear Editor,

Carbon and nitrogen, the uptake and intracellular metabolisms of which are tightly coupled^1^, are the two most fundamental nutrients for all living organisms. As one of the most ancient autotrophic bacteria, cyanobacteria utilize photosynthesis to convert the inorganic carbon (C_i_) into carbohydrates. Carbon fixation is catalyzed by ribulose-1,5-bisphosphate carboxylase/oxygenase (RuBisCO), which is a naturally inefficient enzyme^2^. In response to gradually decreased CO_2_ and elevated O_2_ levels in the atmosphere, cyanobacteria have evolved a unique CO_2_-concentrating mechanism (CCM), which can substantially accumulate CO_2_ in the vicinity of RuBisCO for improved carboxylation efficiency^3^. The cyanobacterial CCM consists of a subcellular self-assembled icosahedral microcompartment, termed the carboxysome, and several C_i_ uptake systems^3^. To date, five C_i_-uptake systems have been identified in cyanobacteria, including three bicarbonate transporters BicA, SbtA, and BCT1, in addition to two CO_2_-uptake complexes: NDH-I_3_ and NDH-I_4_.

SbtA, which is ubiquitous in cyanobacteria, is an inducible high-affinity sodium-dependent HCO_3_^–^ symporter within the TC.2.A.83 family of Na^+^/solute symporters^4,5^. At a low level of intracellular C_i_, SbtA of an up-regulated level can recruit its partner protein SbtB to the membrane^6^. The *sbtB* gene from the *sbtA–sbtB* operon encodes a PII-like signaling protein that senses the intracellular level of the secondary messenger cAMP that correlates with high C_i_ conditions, versus AMP at low C_i_ conditions, thus functioning as a C_i_ sensor in cyanobacteria^6^.

Here we purified *Synechocystis* sp. PCC 6803 SbtA and solved its cryo-EM structure at 3.50 Å resolution (Supplementary Fig. S1). SbtA adopts a trimeric structure, each subunit of which consists of two inverted structural repeats, namely TM1–5 and TM6–10 (Fig. 1a), which are similar to each other in topology but have antiparallel orientations within the membrane (Supplementary Fig. S2). The two structural repeats possess a root-mean-square deviation (RMSD) of 2.63 Å over 135 Cα atoms. At the 3D level, all 10 TMs of each SbtA subunit are folded into two domains, a core domain of six TMs (TM3–5 and TM8–10) and a gate domain of four TMs (TM1–2 and TM6–7) (Fig. 1a; Supplementary Fig. S3). The two domains have a buried interface of ~2000 Å^2^, which is mainly mediated by hydrophobic interactions between residues from TM4–5 to TM9–10 of the core domain and the four TMs of the gate domain (Fig. 1a). Notably, the TM4 and TM9 helices in the core domain are unfolded and form a crossover at the middle. In the gate domain, TM1 and TM6 are divided into two helical moieties, forming a kink at the middle (Fig. 1a; Supplementary Fig. S3). In fact, this feature of discontinuous transmembrane helices is common in previously reported secondary active transporters^7^. Structural analysis showed that SbtA of a 5 + 5 fold is topologically similar to *Neisseria meningitides* ASBT^8^. However, SbtA possesses distinct TM conformations from ASBT, especially those at the gate domain, with an RMSD of 4.4 Å over 249 Cα atoms.

**Fig. 1:**
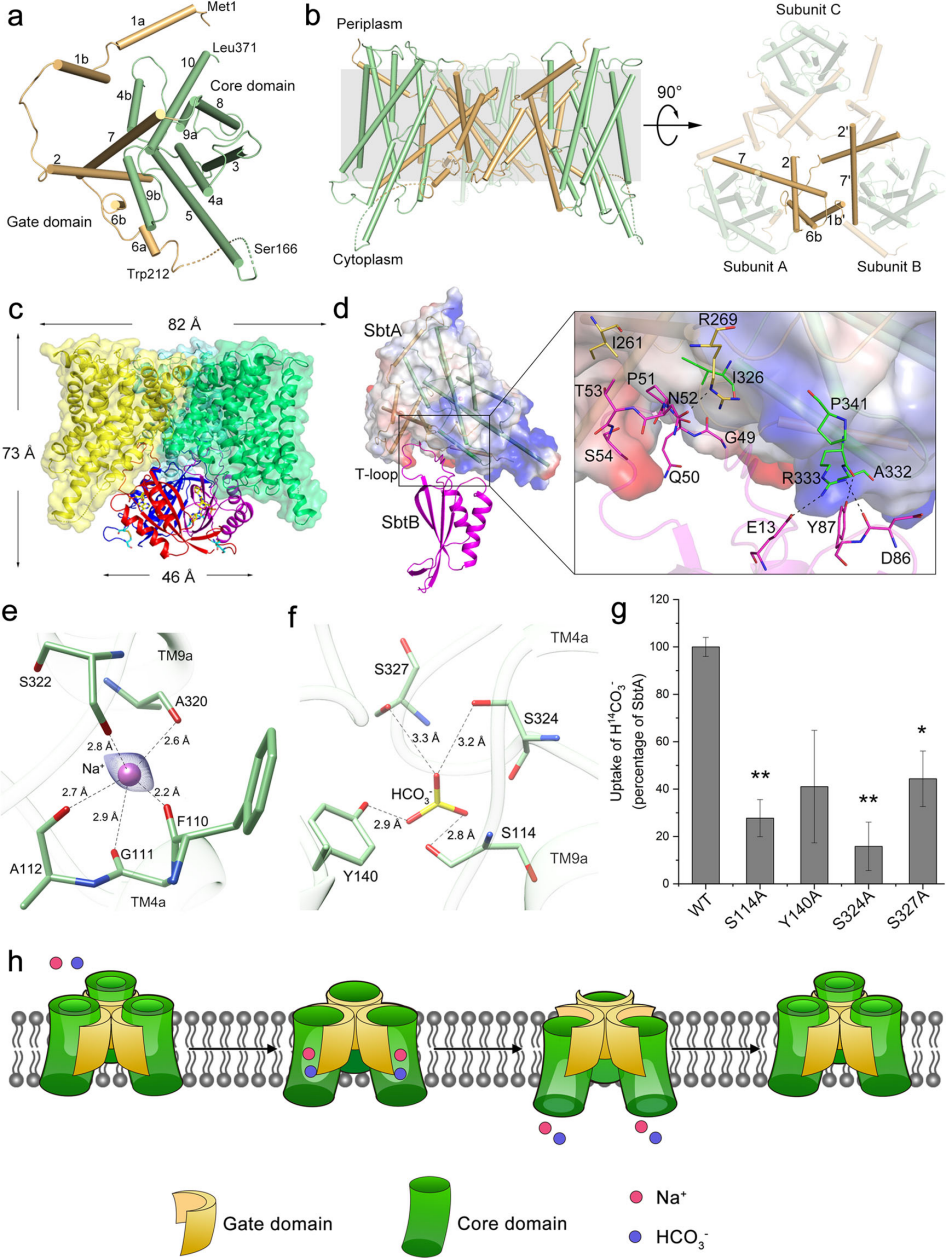
Cryo-EM structures of SbtA and its complex with SbtB. **a** Overall structure of a SbtA subunit viewed from the extracellular side with helices shown as cylinders. **b** Side view of the trimeric structure of SbtA (left) and top view from the intracellular side (right). The gate domains are colored in orange, whereas the core domains are colored in green. The missing residues between Ser166 and Trp212 are indicated as a dashed line. **c** Cartoon representation of SbtA*–*SbtB complex structure. Three subunits of SbtB are colored in magentas, red and blue, respectively. The C-terminal disulfide bond between Cys105 and Cys110 is indicated by blue sticks. An AMP molecule in the nucleotide-binding cleft is shown as yellow sticks. **d** Interfaces between a pair of SbtA and SbtB subunits. The interacting residues are shown as sticks, with the polar interactions indicated by dashed lines. **e** The putative Na^+^-binding site. The Na^+^ is shown as a violet sphere whereas the Na^+^-binding residues are shown as sticks. The polar interactions are indicated by dashed lines. The cryo-EM density map of Na^+^ is shown in blue mesh. **f** The putative HCO_3_^–^-binding site modeled by HADDOCK. The HCO_3_^–^ molecule is shown as sticks and colored by atoms. **g** The HCO_3_^–^ transport activity assays of the wild-type SbtA and mutants in *E. coli* membrane vesicles. Three independent experiments were performed for each assay. The means and standard deviations were calculated and the data are presented as means ± SD. Two-tailed Student’s *t*-test is used for the comparison of statistical significance. The *P* values of < 0.05 and < 0.01 are indicated with * and **, respectively. **h** A proposed elevator mechanism of Na^+^-dependent bicarbonate transport of the trimeric SbtA. The Na^+^ and HCO_3_^–^ are shown as red and blue spheres, respectively. SbtA of an outward-open conformation recruits the substrates HCO_3_^–^ and Na^+^ from the periplasm, accompanied by a rigid-body movement of core domains (green) against the immobile gate domains (yellow). Afterwards, the substrates are released into the cytosol followed by the turnover of SbtA into the resting state.

The tightly packed trimeric structure of SbtA is exclusively stabilized by the gate domains at the center, with a threefold axis perpendicular to the membrane plane (Fig. 1b). The trefoil-like helical bundle of the gate domains possesses a completely buried interface area of ~3000 Å^2^, mainly via hydrophobic interactions. As observable from the extracellular side, the N-terminal moieties of three TM7 helices are bent and interact with each other, whereas from the intracellular side, the N-termini of three TM2 helices form pairwise crossovers via hydrophobic interactions (Fig. 1b). In addition, TM1b of one subunit is positioned against TM6b of the neighboring subunit via hydrophobic interactions, further stabilizing the trimeric structure at the lateral side (Fig. 1b).

It has been reported that when SbtB and SbtA are co-expressed in Escherichia coli (*E. coli*), they form a complex^9^. To elucidate the fine interaction mode between SbtA and SbtB, we purified the SbtA*–*SbtB complex in the presence of AMP and solved its 3.15 Å cryo-EM structure (Supplementary Fig. S4). The SbtB trimer is associated with the intracellular face of the SbtA trimer (Fig. 1c), sharing an overlapped threefold axis. The overall shape of the complex resembles a cylindrical cone of ~73 Å in height, and ~82 and 46 Å in diameter for the SbtA and SbtB trimers, respectively (Fig. 1c). Similar to the previously reported PII and PII-like structures^10^, SbtB also adopts a trimeric structure, each subunit of which shows a canonical PII ferredoxin-like fold with a well-structured T-loop (Fig. 1c; Supplementary Fig. S5). The C-terminal CGPxGC motif, which is conserved in some SbtB homologs, also forms a hairpin structure via a disulfide bond between Cys105 and Cys110 (Fig. 1c; Supplementary Fig. S5). In addition, an AMP molecule binds to the nucleotide-binding cleft between each pair of neighboring SbtB subunits (Fig. 1c; Supplementary Fig. S5).

In the complex, the core structure of SbtB does not directly interact with the intracellular surface of SbtA. Instead, the structured T-loop of SbtB, which extends ~20 Å from the core structure of SbtB, is inserted into the inter-domain cleft of the corresponding SbtA subunit (Fig. 1d), yielding an interface area of ~740 Å^2^. The inter-domain cleft of SbtA is formed by the intracellular parts of TM2 and TM7 of the gate domain, in addition to TM9 and TM10 of the core domain. The extended SbtB T-loop runs along the hydrophobic inter-domain cleft of SbtA, forming extensive hydrophobic interactions (Fig. 1d) via relatively conserved residues (Supplementary Fig. S6). Similar to the complex structure of AmtB*–*GlnK, in which the T-loop of PII protein GlnK inserts deeply into the cytoplasmic pore exit of the ammonia channel AmtB^11^, the SbtA*–*SbtB structure showed that SbtB T-loop partially blocks the substrate tunnel exit of SbtA, indicating that SbtB might attenuate the transport activity of SbtA under certain physiological conditions. Consistently, the previous results showed that SbtB inhibited the SbtA-mediated HCO_3_^–^ uptake in *E. coli*^9^. However, a recent report demonstrated that the light-regulated SbtA function in vivo is independent of SbtB in *Synechococcus* PCC 7942^12^. Thus the fine in vivo regulatory mechanism of SbtA*–*SbtB remains unsolved, but might be involved in the coordinated regulation of light*–*dark transitions, the intracellular homeostasis of adenyl-nucleotides and redox status.

At the peripheral side of TM crossover within the center of SbtA core domain in the SbtA*–*SbtB complex, there is a clear cryo-EM density most likely corresponding to a sodium ion (Fig. 1e), given SbtA is a Na^+^-dependent transporter. In each subunit, the Na^+^ forms five coordinate bonds with the main-chain oxygens of residues Phe110, Gly111, and Ala112 from TM4a and Ala320 and Ser322 from TM9a (Fig. 1e). Notably, in the previously reported Na^+^-dependent transporters^8,13^, Na^+^ and its co-transported substrate are located nearby the crossover of TMs. We thus docked HCO_3_^–^ in the vicinity of the crossover of the complexed SbtA via HADDOCK^14^. In this docking model, the HCO_3_^–^-binding site is located at the interface of the core and gate domains. It binds to a cavity formed by the other side of the crossover and forms four hydrogen bonds with Ser114, Tyr140, Ser324, and Ser327 (Fig. 1f). Sequence analysis revealed that most of these putative substrate-binding residues are conserved among SbtA homologs (Supplementary Fig. S7). Furthermore, mutating any of these putative HCO_3_^–^-binding residues to Ala led to a dramatic decrease of HCO_3_^–^ transport activity of SbtA in *E. coli* membrane vesicles to about 10%–40% of the wild-type level (Fig. 1g). The results demonstrated that these residues are necessary for optimal transport activity of SbtA.

Structural analysis revealed that the substrate-binding sites of free SbtA are inaccessible to the solvent from either intracellular or extracellular side. Thus, the structure of free SbtA represents an occluded conformation. Respectively superimposing the two individual domains of each SbtA subunit against those in the SbtB-bound form showed little structural variations (Supplementary Fig. S8a). However, upon binding to SbtB, the two domains of each SbtA subunit undergo a significant rigid-body movement against each other. Given the superimposed gate domains, each core domain of the complexed SbtA tilts about 10° against the trefoil-like center, accompanied by a 4-Å slide of the core domain toward the intracellular space (Supplementary Fig. S8b). As a result, the two domains of SbtA in SbtB-bound form are relatively separated from each other, yielding an inward-open conformation (Supplementary Fig. S8b). The present structures of SbtA in the free and SbtB-bound forms capture two snapshots in the transport cycle which enable us to propose a putative model for SbtA-mediated HCO_3_^–^ transport (Fig. 1h). Initially, SbtA was assumed to adopt an outward-open conformation ready to uptake the substrates HCO_3_^–^ and Na^+^. Substrate binding most likely promotes SbtA to adopt an occluded conformation, as seen in our free SbtA structure with a Na^+^ bound in the pocket. Afterwards, the core domains of SbtA slide toward the cytosolic side against the immobile gate domains, resulting in an inward-open conformation of SbtA. Accordingly, the substrate tunnel is open toward the intracellular space (Supplementary Fig. S8b). Finally, the substrates are released to the cytosol, accompanied by the turnover of SbtA to the resting state for the next cycle of transport. This transport model of SbtA is reminiscent of the elevator alternating-access transport mechanism, in which the substrate-binding domain moves along the relatively immobile scaffold domain^15^.

In summary, this study provides structural insights into the cyanobacterial high-affinity HCO_3_^–^ transporter SbtA, which exhibits a new architecture of Na^+^/solute symporters and its fine interaction pattern with a PII-like protein SbtB. Structural analysis combined with site-directed mutagenesis enabled us to identify the substrate-binding pocket and propose an elevator transport mechanism of SbtA.

## Supplementary information

Supplementary Information

## Data Availability

The cryo-EM structures of SbtA and SbtA*–*SbtB have been deposited at PDB under the codes of 7CYE and 7CYF, respectively. The cryo-EM density maps of SbtA and SbtA*–*SbtB have been deposited at the Electron Microscopy Data Bank (EMD-30498 for SbtA and EMD-30499 for SbtA*–*SbtB).
