# Supplementary material for: Structures of cyanobacterial bicarbonate transporter SbtA and its complex with PII-like SbtB
Source: Cell Discov. 2021 Aug 10;7:63. doi: 10.1038/s41421-021-00287-w (PMC8352866; doi:10.1038/s41421-021-00287-w)
Supplement: Supplementary file 1 — Supplementary Information [file 41421_2021_287_MOESM1_ESM.pdf]

Supplementary Information for

Structures of cyanobacterial bicarbonate transporter SbtA and its complex with PII-like SbtB

Xiao-Yu Liu, Wen-Tao Hou, Liang Wang, Bo Li, Yu Chen, Yuxing Chen, Yong-Liang Jiang*, Cong-Zhao Zhou*

This file includes:

Materials and methods

Supplementary Figs. S1 to S8

Supplementary Table S1

Materials and methods

Protein expression and purification

The codon-optimized genes *sbtA* and *sbtA-sbtB* were synthesized by Genewiz Biotech and cloned into pET-28a (YouBio) carrying an N-terminal 6×His-tag on SbtA. The plasmids were transformed into *E. coli* C43 (WeidiBio), growing at 37°C in Luria Bertani (LB) culture medium, supplemented with 30 μg mL^{-1} kanamycin. Protein expression was induced by adding 0.4 mM isopropyl-β-D-thiogalactoside (IPTG, BioFroxx) when the $OD_{600\ nm}$ reached 1.1~1.3. After incubation for another 4 hr at 37°C, the cells were collected and resuspended in the lysis buffer containing 25 mM Tris-HCl pH 8.0, 300 mM NaCl, 5% glycerol and stored at -80°C before use.

For purification of SbtA, the collected cells were resuspended in the lysis buffer and lysed by AH-1500 High Pressure Homogeniser (ATS, inc.) with 5 passes at 700~800 bar. Cell debris was removed by centrifugation at 17,300 × g for 20 min. The supernatant was ultra-centrifuged at 200,000 × g for 1 hr. The membrane was collected and solubilized by adding 1% (w/v) dodecyl-β-D-maltopyranoside (DDM, Bluepus) and 1% (w/v) Lauryl Maltose Neopentyl Glycol (LMNG, Anatrace) for 1 hr at 4°C in the lysis buffer. After ultracentrifugation at 200,000 × g for 0.5 hr, the supernatant was loaded onto a Ni-NTA resin (GE Healthcare), washed with the buffer containing 25 mM Tris-HCl pH 8.0, 300 mM NaCl, 5% glycerol, 40 mM imidazole, 0.02% (w/v) glyco-diosgenin (GDN, Anatrace) and then eluted by the same buffer supplemented with 300 mM imidazole. The eluate was then concentrated to about 1 mL by concentrators with a relative molecular mass cut-off of 100 KDa, and was applied to a Superdex 200

Increase 10/300 gel filtration column (GE Healthcare) equilibrated in the buffer containing 25 mM Tris-HCl pH 8.0, 150 mM NaCl, 5% glycerol, 0.02% (w/v) GDN. The peak fractions were collected for further procedures.

Expression and purification of SbtA-SbtB complex were performed following the same protocol as SbtA, except that all purification buffers were supplemented with 2 mM AMP and 5 mM $NaHCO_3$.

Cryo-EM sample preparation

Aliquots of 3.5 µL SbtA and SbtA-SbtB samples (~6 mg/mL) were applied to the glow-discharged grids (Quantifoil holey carbon Cu R1.2/1.3 grid). The glow-discharged grid was pretreated with 0.01% (w/v) polylysine to improve the adhesion of SbtA proteins to the carbon grid[1]. Then grids were blotted for 4.5 sec and plunge-frozen in liquid ethane vitrified by liquid nitrogen using Vitrobot Mark IV (FEI Company) at 8°C and 100% humidity.

Cryo-EM data collection and processing

The cryo-EM grids of SbtA were loaded into a Titan Krios transmission electron microscope (ThermoFisher Scientific) operating at 300 KeV with a Gatan K2 Summit direct electron detector at the Center for Integrative Imaging of Hefei National Laboratory for Physical Sciences at the Microscale, University of Science and Technology of China (USTC). Totally 5,587 movie stacks were collected in a super-resolution mode with a defocus range from -1.5 to -2.0 µm. Each movie stack of 32

frames was exposed for 6.4 sec under a dose rate of 10 e/pixel/sec, resulting in a total dose of ~60 e Å^{-2}. All movie stacks were motion-corrected and dose-weighted using MotionCor2[2], and were binned two-fold to yield a pixel size of 1.01 Å. The contrast transfer function (CTF) estimation was performed by CTFFIND4[3] within RELION-3.1[4], which was used for further processing. In total 1,394,695 particles were auto-picked and extracted at a 2-fold binned with a pixel size of 2.02 Å. After multi-rounds of 2D classification, 407,068 particles were used for 3D classification, searching for 4 classes using an initial reference generated by the 3D initial model within RELION-3.1[4]. 234,137 particles from the best class were re-extracted at a pixel size 1.01 Å without binning and used for 3D refinement yielding a 3.8 Å map. After multi-rounds of 3D classification with skip alignment, 110,385 particles were selected for final 3D refinement yielding a 3.7 Å map. After the CTF refinement and Bayesian polishing, the final map gave a resolution of 3.50 Å (Supplementary Fig. S1).

The cryo-EM data of SbtA-SbtB complex were collected at the Center for Biological Imaging at the Institute of Biophysics (IBP), Chinese Academy of Sciences. A total of 4,703 cryo-EM movie stacks, each of which comprises 40 frames, were collected using the similar parameters as that for SbtA, yielding a pixel size of 1.04 Å. After motion correction by MotionCor2[2] and CTF estimation performed using CTFFIND4[3]. A total of 1,332,746 particles were auto-picked and extracted at a 2-fold binned pixel size of 2.08 Å. After multi-rounds of 2D classification, 759,355 particles were selected for further 3D classification with 4 classes using the reference generated by the 3D initial model. 538,435 particles from the best class were refined and re-

extracted for further 3D refinement yielding a 3.35 Å map. Then multi-rounds of 3D classification with skip alignment were performed. Finally, 41,547 particles from one of the classes were further refined and post-processed to yield a 3.15 Å map (Supplementary Fig. S4). Map resolution was estimated with the gold-standard Fourier shell correlation 0.143 criterion[5]. Local resolutions were estimated using Resmap[6] with RELION-3.1[4].

Model building and refinement

The cryo-EM density of SbtA showed clear features which enable us to build the atomic models *ab initio*. First, we built an all-alanine model manually into the map using Coot[7]. Then assign of the residues was facilitated by the secondary structure prediction with the assistance by the bulky residues. After several rounds of manual building, the model was almost completed built and automatically refined against the map by phenix.real_space_refine program in PHENIX[8] with secondary structure and geometry restraints. The initial model of SbtA-SbtB complex was built by fitting the SbtA and SbtB (PDB: 5O3R) structures into the map using the UCSF Chimera[9]. Due to drastic structural variations of two domains of SbtA, we individually fit each of two domains of SbtA into the SbtA-SbtB map. Then model building and refinement was accomplished manually by Coot[7]. The final structures showed good geometry and were further evaluated using MolProbity[10]. All structure figures were prepared in PyMOL (https://pymol.org). A list of parameters of cryo-EM data collection, processing, structure determination and refinement is provided in the Supplementary Table S1.

Preparation of membrane vesicles

The right-side-out membrane vesicles were prepared according to the previous report[11]. Briefly, the process was started with the preparation of osmotically sensitive *E. coli* cells. The empty plasmid or plasmids carrying SbtA or SbtA-SbtB were transformed into *E. coli* C43. Then cells were cultured in M63 medium (2 g $(NH_4)_2SO_4$, 13.6 g KH_2PO_4, 0.5 mg $FeSO_4 \cdot 7H_2O$, 0.246 g $MgSO_4 \cdot 7H_2O$, 4.2 g KOH, 0.2% glucose, 0.1% casamino acids and 0.1 ml 0.5% vitamin B_1 per liter) and induced at 37°C for 4 hr supplemented with 0.4 mM IPTG. The culture was centrifuged at 16000 × g to harvest the cells and the pellet was washed twice with 10 mM Tris-HCl, pH 8.0 on ice. The cells were weighted and resuspended with 30 mM Tris-HCl, pH 8.0, and 20% sucrose, and flash-frozen with liquid nitrogen.

Next, the thawed cells were diluted with 30 mM Tris-HCl, pH 8.0, and 20% sucrose (1g wet weight, per 80 mL) supplemented with 10 mM EDTA-KOH, pH 7.0 and 0.5 mg/mL lysozyme, and swirled for 30 min by means of a magnetic stirrer at room temperature. The protoplast suspensions were centrifuged at 16,000 × g for 15 min and the pellet was resuspended in 10 mL buffer containing 0.1M KH_2PO_4/K_2HPO_4, pH 6.6, 20 mM $MgSO_4$ and homogenized by ULTRA-TURRAX (IKA). Then the suspension was poured directly into 300-fold volumes of 50 mM KH_2PO_4/K_2HPO_4, pH 6.6 supplemented with 100 μg/mL DNase and 100 μg/mL RNase. The lysate was incubated for 15 min at 37°C with vigorous swirling. Then 10 mM EDTA-KOH was supplemented and the lysate was incubated for another 15 min. Afterwards, 15 mM

$MgSO_4$ was supplemented in the lysate. In the end, the lysate was centrifuged at 16,000 g for 30 min and the pellet was centrifuged at 45,000 × g for 30 min to isolate the membranes.

The isolated membranes were homogenized in a solution of 0.1 M KH_2PO_4/K_2HPO_4, pH 6.6, containing 10 mM EDTA on ice. Then the suspension was centrifuged at 800 × g until the supernatant fluid was clear. The pellet was washed 4~6 times by centrifugation at 45,000 × g for 30 min in the solution of 0.1 M KH_2PO_4/K_2HPO_4, pH 6.6, containing 10 mM EDTA on ice. Finally, the obtained membrane vesicles were resuspended by homogenization in the transport assay buffer[12] (50 mM CHES-KOH pH 9.0, 0.3 mM $MgSO_4$, 0.26 mM $CaCl_2$, 0.22 mM K_2HPO_4) at a concentration of 5 mg/mL (wet weight) and frozen in small aliquots in liquid nitrogen and stored in -80°C before use. The amounts of proteins in the membrane vesicles for each preparation were quantified by purifying the proteins from the same batch of cells by Coomassie brilliant blue staining.

Transport activity assays

For each assay sample, 50 μL of membrane vesicles was thawed. The substrates of 12 mM NaCl and 0.1 nCi ^{14}C labeled $NaHCO_3$ (~12 μM, American Radiolabled Chemicals) were added in the system supplemented with the transport assay buffer to 100 μL. All reactions were performed in 30°C for 30 sec and terminated by rapid filtration on a glass filter (25 mm GF/F, Whatman) by suction, followed by immediate washing the filter with 10 mL of the washing buffer (the transport assay buffer

supplemented with 120 mM $NaHCO_3$ to prevent back flow). The filters were soaked in 3 mL ULTIMA Gold (PerkinElmer) overnight before liquid scintillation counting. The vesicles prepared with induced *E. coli* transformed by the empty plasmid pET-28a were tested as the control group.

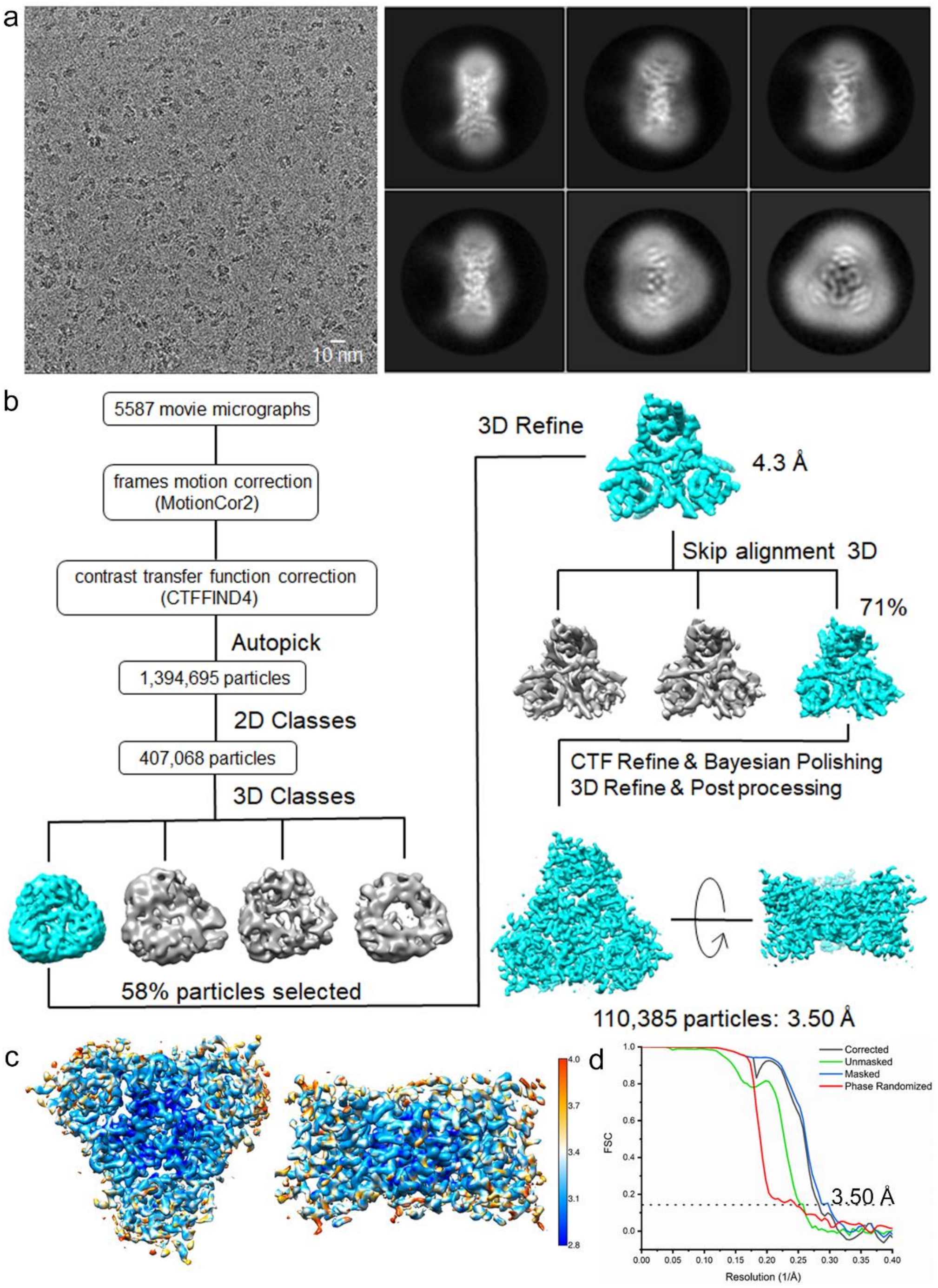

Supplementary Fig. S1 Cryo-EM analysis of SbtA in *Synechocystis* sp. PCC 6803. **a** Representative cryo-EM micrograph of SbtA and 2D classes. **b** Data processing flowchart with particle distributions. **c** Resmap resolution slice and resolution map for SbtA, shown in the top view and the side view respectively. **d** Fourier shell correlation (FSC) curves showing a resolution of 3.50 Å.

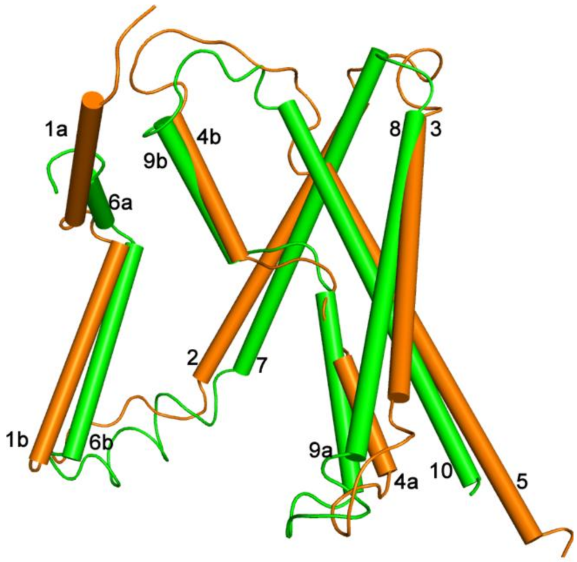

Supplementary Fig. S2 Superposition between TM1–5 and TM6–10 of SbtA.

TM1–5 and TM6–10 are colored in orange and green, respectively.

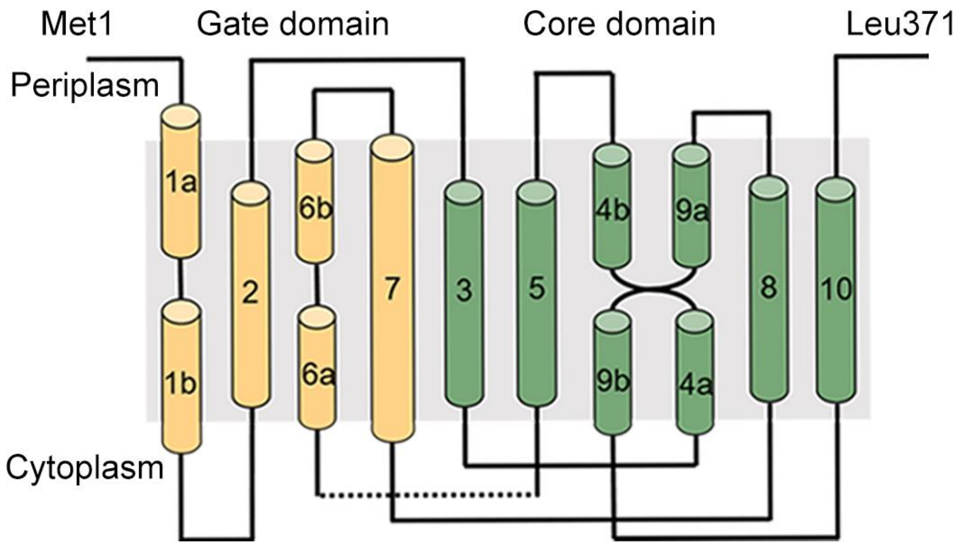

Supplementary Fig. S3 Topology of a SbtA subunit. The gate domain and core domain of SbtA are colored in orange and green, respectively.

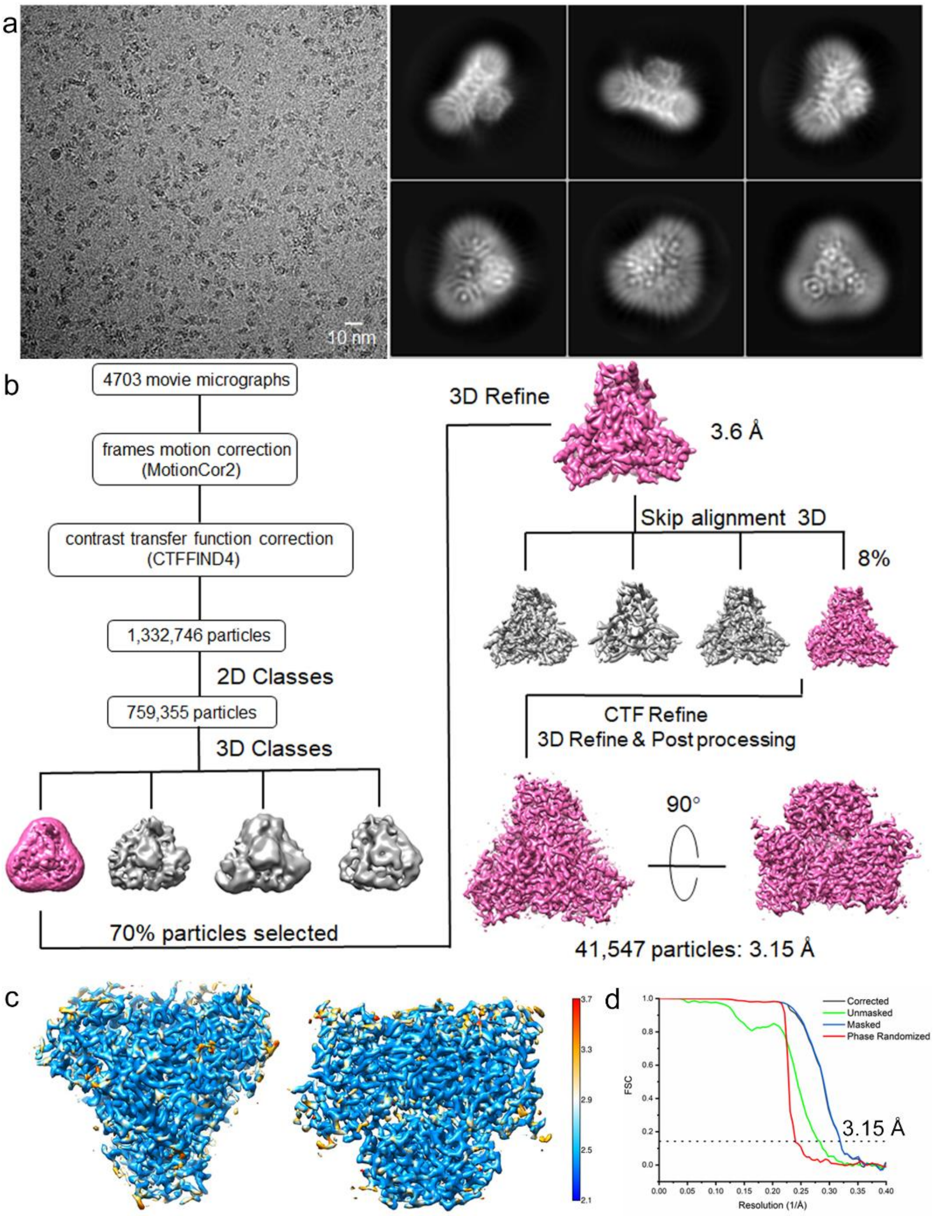

Supplementary Fig. S4 Cryo-EM analysis of the SbtA-SbtB complex in *Synechocystis* sp. PCC 6803. a Representative cryo-EM micrograph of SbtA-SbtB and 2D classes. **b** Data processing flowchart with particle distributions. **c** Resmap resolution slice and resolution map for SbtA-SbtB, shown in the top view and side view respectively. **d** FSC curves showing a resolution of 3.15 Å.

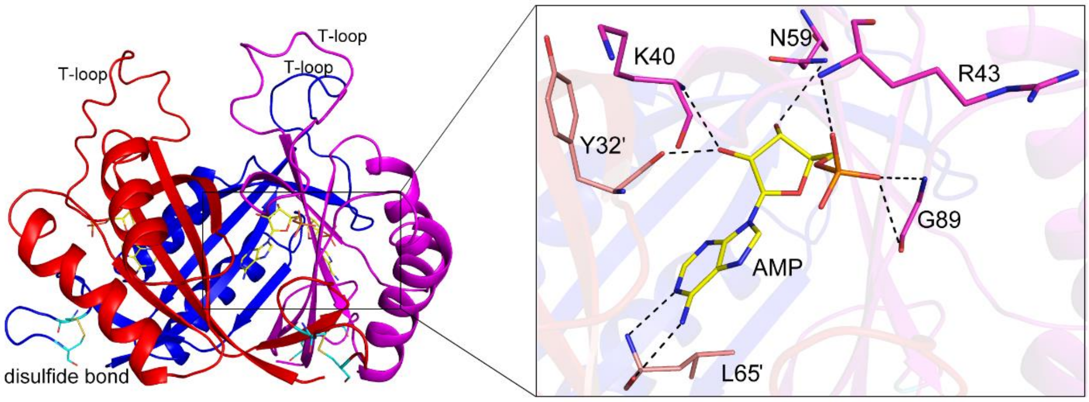

Supplementary Fig. S5 The AMP-binding site of SbtB. The SbtB trimer is shown as cartoon on the left, with the AMP-binding pocket zoomed-in on the right. The AMP molecule and the interacting residues are shown as sticks. The polar interactions are indicated by dashed lines.

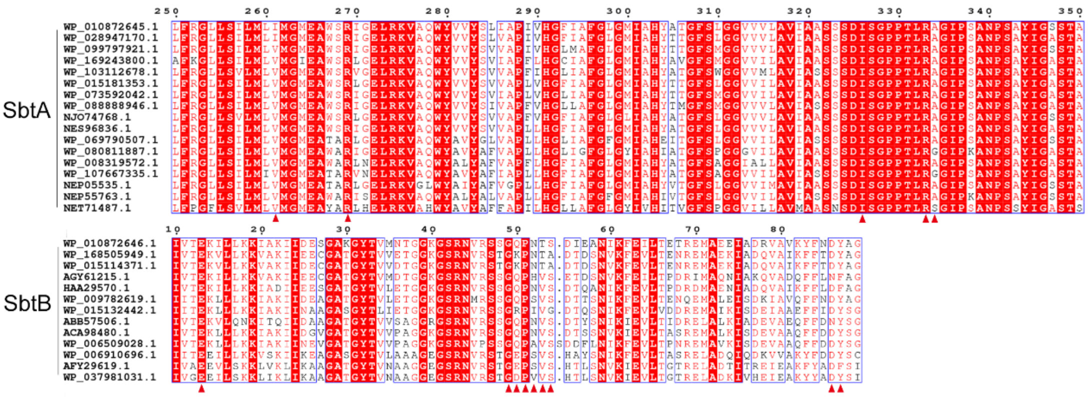

Supplementary Fig. S6 Multiple-sequence alignment of the interacting residues between SbtA and SbtB by MultAlin. The interacting residues are indicated by red triangles. The NCBI accession codes for SbtA homologs sequences are: WP_010872645.1 (*Synechocystis* sp. PCC 6803), WP_028947170.1 (*Synechocystis* sp. PCC 6714), WP_099797921.1 (*Synechococcus lividus*), WP_169243800.1 (*Chroococcidiopsis* sp. PCC 6712), WP_103112678.1 (*Microcystis aeruginosa*), WP_015181353.1 (*Microcoleus* sp. PCC 7113), WP_073592042.1 (*Phormidium ambiguum*), WP_088888946.1 (*Leptolyngbya ohadii*), NJO74768.1 (*Leptolyngbyaceae cyanobacterium* RM1_406_9), NES96836.1 (*Desertifilum* sp. SIO1I2), WP_069790507.1 (*Cyanobacterium* sp. IPPAS B-1200), WP_080811887 (*Halomicronema hongdechloris*), WP_008319572.1 (*Leptolyngbya* sp. PCC 6406), WP_107667335.1 (*Cyanothece* sp. BG0011), NEP05535.1 (*Okeania* sp. SIO4D6), NEP55763.1 (*Symploca* sp. SIO2G7), NET71487.1 (*Sphaerospermopsis* sp. SIO1G2). The NCBI accession codes for SbtB homologs sequences are: WP_010872646.1 (*Synechocystis* sp. PCC 6803), WP_168505949.1 (*Anabaena* sp. UHCC 0187), WP_015114371.1 (*Nostoc* sp. PCC 7107), AGY61215.1 (*Microcystis aeruginosa* CCAP 1450), HAA29570.1 (*Cyanobacteria* UBA8553), WP_009782619.1 (*Lyngbya*

sp. PCC 8106), WP_015132442.1 (*Leptolyngbya* sp. PCC 7376), ABB57506.1 (*Synechococcus elongatus* PCC 7942), ACA98480.1 (*Synechococcus* sp. PCC 7002), WP_006509028.1 (*Xenococcus* sp. PCC 7305), WP_006910696.1 (*Cyanobium* sp. PCC 7001), AFY29619.1 (*Cyanobium* sp. PCC6307), WP_037981031.1 (*Synechococcus* WH5701).

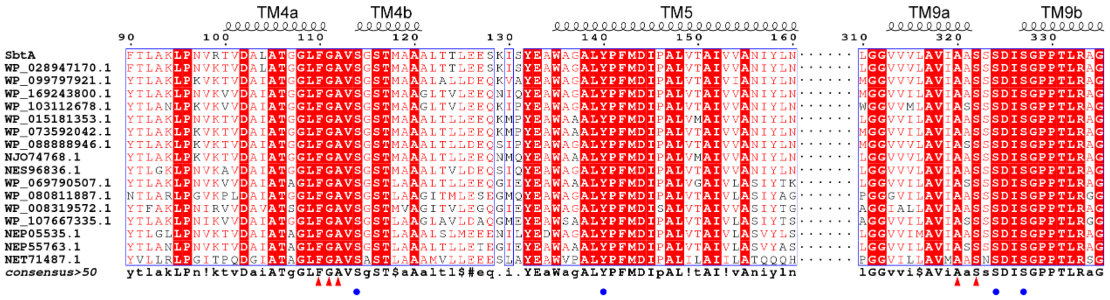

Supplementary Fig. S7 Multiple-sequence alignment of residues in TM4, TM5 and TM9 of SbtA homologs by MultAlin. The putative Na^{+}- and HCO_3^{-}-binding residues of SbtA are indicated by red triangles and blue dots, respectively. The NCBI accession codes for SbtA homologs sequences are: SbtA (*Synechocystis* sp. PCC 6803), WP_028947170.1 (*Synechocystis* sp. PCC 6714), WP_099797921.1 (*Synechococcus lividus*), WP_169243800.1 (*Chroococcidiopsis* sp. PCC 6712), WP_103112678.1 (*Microcystis aeruginosa*), WP_015181353.1 (*Microcoleus* sp. PCC 7113), WP_073592042.1 (*Phormidium ambiguum*), WP_088888946.1 (*Leptolyngbya ohadii*), NJO74768.1 (*Leptolyngbyaceae cyanobacterium* RM1_406_9), NES96836.1 (*Desertifilum* sp. SIO1I2), WP_069790507.1 (*Cyanobacterium* sp. IPPAS B-1200), WP_080811887 (*Halomicronema hongdechloris*), WP_008319572.1 (*Leptolyngbya* sp. PCC 6406), WP_107667335.1 (*Cyanothece* sp. BG0011), NEP05535.1 (*Okeania* sp. SIO4D6), NEP55763.1 (*Symploca* sp. SIO2G7), NET71487.1 (*Sphaerospermopsis* sp. SIO1G2).

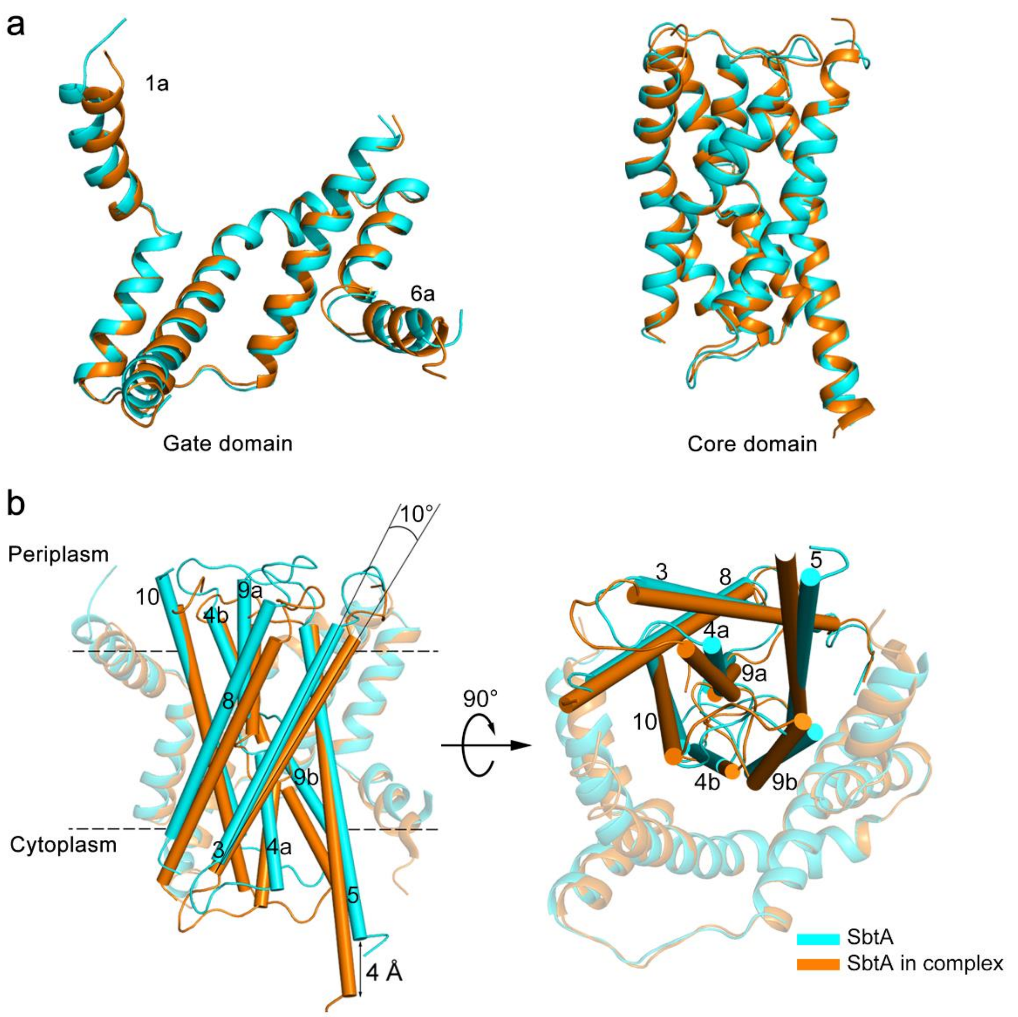

Supplementary Fig. S8 Comparison of SbtA structures in the free and complexed forms. a Comparison of the gate domain and the core domain of one SbtA subunit, respectively. **b** Conformational changes of SbtA upon binding to SbtB, shown in the top view and the side view respectively. The free and SbtB complexed SbtA are colored in cyan and orange, respectively.

Supplementary Table S1. Cryo-EM parameters, data collection and refinement statistics.

	SbtA (EMD-30498) (PDB 7CYE)	SbtA-SbtB (EMD-30499) (PDB 7CYF)
Data collection and processing		
Magnification	29,000	130,000
Voltage (keV)	300	300
Electron exposure (e^-/Å^2)	60	60
Defocus range (μm)	1.5 to 2.0	1.5 to 2.0
Pixel size (Å)	1.01	1.04
Symmetry imposed	C3	C3
Initial particle images (no.)	1,394,695	1,332,746
Final particle images (no.)	110,385	41,547
Map resolution (Å)	3.50	3.15
FSC threshold	0.143	0.143
Map resolution range (Å)	2.02~999	2.08~999
Refinement		
Real-space correlation coefficient	0.80	0.80
Initial model used (PDB code)	*ab initio*	7CYE, 5O3R
Map sharpening B factor (Å^2)	-145.44	-125.174
Model composition		
Non-hydrogen atoms	7,266	9,993
Protein residues	978	1,329
Waters	0	0
RMS deviation from ideality		
Bond lengths (Å)	0.010	0.008
Bond angles (°)	1.364	1.204
Validation		
MolProbity score	1.79	1.64
Clash score	3.89	3.24
Poor rotamers (%)	0.78	0.57
Ramachandran statistics		
Favored regions (%)	87.37	90.85
Allowed regions (%)	12.63	9.15
Outliers (%)	0.00	0.00

References

1 Woodcock, C., Frado, L. L., Green, G. & Einck, L. Adhesion of particulate specimens to support films for electron microscopy: a model system for assessing the surface properties of support films, and its application to chromatin particles. *J. microsc.* **121**, 211-220 (1981).

2 Zheng, S. Q. *et al.* MotionCor2: anisotropic correction of beam-induced motion for improved cryo-electron microscopy. *Nat. Methods* **14**, 331-332 (2017).

3 Rohou, A. & Grigorieff, N. CTFFIND4: Fast and accurate defocus estimation from electron micrographs. *J. Struct. Biol.* **192**, 216-221 (2015).

4 Zivanov, J., Nakane, T. & Scheres, S. H. W. Estimation of high-order aberrations and anisotropic magnification from cryo-EM data sets in *RELION*-3.1. *IUCrJ* **7**, 253-267 (2020).

5 Rosenthal, P. B. & Henderson, R. Optimal determination of particle orientation, absolute hand, and contrast loss in single-particle electron cryomicroscopy. *J. Mol. Biol.* **333**, 721-745, (2003).

6 Kucukelbir, A., Sigworth, F. J. & Tagare, H. D. Quantifying the local resolution of cryo-EM density maps. *Nat. Methods* **11**, 63-65 (2014).

7 Emsley, P. & Cowtan, K. Coot: model-building tools for molecular graphics. *Acta Crystallogr. D* **60**, 2126-2132 (2004).

8 Adams, P. D. *et al.* PHENIX: a comprehensive Python-based system for macromolecular structure solution. *Acta Crystallogr. D* **66**, 213-221 (2010).

9 Pettersen, E. F. *et al.* UCSF Chimera--a visualization system for exploratory research and analysis. *J. Comput. Chem.* **25**, 1605-1612 (2004).

10 Davis, I. W. *et al.* MolProbity: all-atom contacts and structure validation for proteins and nucleic acids. *Nucleic Acids Res.* **35**, W375-383 (2007).

11 Kaback, H. R. Bacterial membranes. *Methods Enzymol.* 22, 99–120 (1971)

12 Shibata, M. *et al.* Genes essential to sodium-dependent bicarbonate transport in cyanobacteria: function and phylogenetic analysis. *J. Biol. Chem.* **277**, 18658-18664 (2002).
